# Remapping of Adult-Born Neuron Activity during Fear Memory Consolidation in Mice

**DOI:** 10.3390/ijms22062874

**Published:** 2021-03-12

**Authors:** Pablo Vergara, Deependra Kumar, Sakthivel Srinivasan, Iyo Koyanagi, Toshie Naoi, Sima Singh, Masanori Sakaguchi

**Affiliations:** 1 International Institute for Integrative Sleep Medicine (WPI-IIIS), University of Tsukuba, Tsukuba, Ibaraki 305-8577, Japan; kumar.deependra.ff@u.tsukuba.ac.jp (D.K.); srinivasan.sakthi.gp@u.tsukuba.ac.jp (S.S.); koyanagi.iyo.su@alumni.tsukuba.ac.jp (I.K.); naoi.toshie.fw@un.tsukuba.ac.jp (T.N.); singh.sima@gmail.com (S.S.); 2 Doctoral Program in Biomedical Sciences, Graduate School of Comprehensive Human Sciences, University of Tsukuba, Tsukuba, Ibaraki 305-8577, Japan; 3 Doctoral Program in Neuroscience, Degree Programs in Comprehensive Human Sciences, Graduate School of Comprehensive Human Sciences, University of Tsukuba, Tsukuba, Ibaraki 305-8577, Japan

**Keywords:** hippocampus, adult neurogenesis, memory, contextual fear conditioning, calcium imaging, sleep

## Abstract

The mammalian hippocampal dentate gyrus is a unique memory circuit in which a subset of neurons is continuously generated throughout the lifespan. Previous studies have shown that the dentate gyrus neuronal population can hold fear memory traces (i.e., engrams) and that adult-born neurons (ABNs) support this process. However, it is unclear whether ABNs themselves hold fear memory traces. Therefore, we analyzed ABN activity at a population level across a fear conditioning paradigm. We found that fear learning did not recruit a distinct ABN population. In sharp contrast, a completely different ABN population was recruited during fear memory retrieval. We further provide evidence that ABN population activity remaps over time during the consolidation period. These results suggest that ABNs support the establishment of a fear memory trace in a different manner to directly holding the memory. Moreover, this activity remapping process in ABNs may support the segregation of memories formed at different times. These results provide new insight into the role of adult neurogenesis in the mammalian memory system.

## 1. Introduction

New neurons are continuously generated in the adult hippocampal dentate gyrus [1]. This adult neurogenesis produces granular neurons that are similar to developmentally born granular neurons [2,3,4]. However, during their maturation, young adult-born neurons (ABNs) show properties that are distinct from those of developmentally born neurons, including higher synaptic plasticity and excitability [3,5,6,7,8] and lower input selectively and inhibitory inputs [9,10,11,12]. These properties allow ABNs to be preferentially activated by novel stimuli [8,13,14] and could allow transitions in active cohorts of ABNs over time to achieve temporal separation and integration of different memories [12,15]. In this case, the time course of memory processing would match that of ABN maturation (i.e., several days or weeks), although the hippocampus also engages in memory processing with shorter time scales (i.e., minutes or hours). For example, sparse ABN activity during rapid eye movement (REM) sleep is necessary during the 6 h after learning but not later [16]. However, the mechanisms by which ABNs contribute to memory processing across time are still largely unknown.

Previous studies have indicated that dentate gyrus neurons are necessary and sufficient for holding a fear memory trace (or engram) [17,18]. However, ABNs may promote both memory separation and memory generalization [11,12], prompting the question of whether ABNs actually hold memory traces. If so, a specific ABN population would be expected to activate during learning and then reactivate during memory retrieval [19]. Although we previously revealed the activity of individual ABNs during learning and retrieval [16], the activity of populations of ABNs during memory consolidation has not yet been characterized.

Here, we reanalyzed our previously published ABN activity using a fear conditioning paradigm [16] at the population level. We provide evidence that a population of ABNs is activated upon fear learning. However, this population does not overlap with that which is active during fear memory retrieval. Moreover, we found that ABNs undergo activity remapping across time during the memory consolidation period. This remapping could allow ABNs to segregate memories encoded not only several days apart [11,12] but also hours apart.

## 2. Results

**Figure 1:**
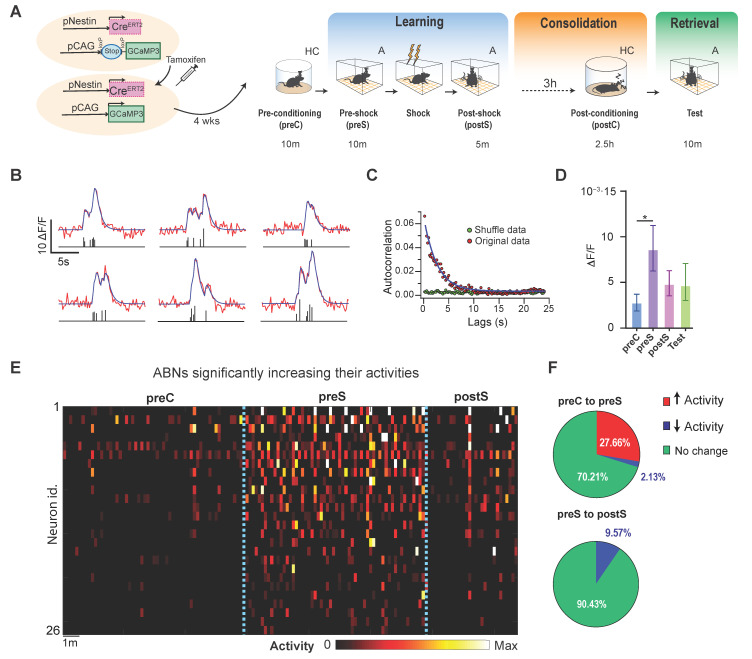
Adult-born neuron (ABN) activity increases during novel context exposure. (**A**) Experimental design. (**B**) Six examples of Ca^2+^ trace deconvolution. Raw signal (red, top) was decomposed into unitary Ca^2+^ events with amplitude (height) and timing information (bottom, black bars). Overlaying blue traces (top) show reconstructed Ca^2+^ signal. (**C**) Circular autocorrelation function of mean activity from actual data (red dots) and temporally shuffled data (green dots). The blue line is an exponential fit. (**D**) Mean unitary event activity in each session. Bootstrap, * *p* < 0.05; *n* = 94 neurons from 4 mice; error bars, 95% confidence interval. (**E**) Activity heatmap shows increased ABN activity in the preshock (preS) period. Block bootstrap; * *p* < 0.05; each time bin, 10 s. (**F**) Pie chart of ABN changing their activities from preconditioning (preC) to preS, and preS to postshock (postS) periods (red, increase; blue, decrease; green, no change).

We reanalyzed the Ca^2+^ imaging data obtained in a previous report [16]. Briefly, we reanalyzed the activity of ~4-week-old ABNs in pNestinCreERT2/pCAG-LSL-GCaMP3 mice throughout the course of a contextual fear conditioning paradigm [16] at the population level. Recordings were performed over three major consecutive periods: learning, consolidation, and retrieval (Figure 1A). Immediately after a recording session in a familiar environment (preconditioning in the home cage, preC; 10 min), the learning period included recording sessions in a conditioning context before foot shock (preshock in context A, preS; 10 min), and the same context after foot shock (postshock in context A, postS; 5 min). In the consolidation period, mice stayed in their home cage for a total of 5.5 h, and recording was performed in the final 2.5 h. During this period, memory consolidation depends on the sparse activity of ABNs during REM sleep [16]. In the postconsolidation period, mice were re-exposed to context A (test; 10 min), during which they exhibited context-specific freezing behavior [16].

Previous reports indicate that ABN Ca^2+^ transients occur at very low rates (~1 transient/min) [13,16]. However, each Ca^2+^ transient usually shows polyphasic dynamics (i.e., contains multiple peaks) (Figure 1B), reflecting the integration of several unitary Ca^2+^ events. This characteristic was not considered in our previous analysis, in which each transient was treated as one event [16]. Therefore, in the present reanalysis, the amplitude and timing of the unitary events comprising each transient were estimated by the deconvolution of raw Ca^2+^ traces (Figure 1B) [20]. This approach did not change our previous conclusions [16]: The ABNs active during REM sleep were predominantly active during the postS period (Figure S1A). Likewise, the ABNs active during the postS, but not those active during retrieval, were predominantly active during REM sleep (Figure S1B). To examine the temporal aggregation of unitary Ca^2+^ events, we calculated a mean circular autocorrelation function (Figure 1C), which provides an estimate of the likelihood of observing one event soon after another [21]. The autocorrelation function assumes a constant value when events are randomly distributed in time, as occurs when events are temporally shuffled (Figure 1C, green). By contrast, the autocorrelation function for actual events showed an exponential decay within 10 s (Figure 1C, red), suggesting that ABN activity is temporally aggregated.

We found that neuron activity increased when mice explored the fear conditioning context (Figure 1D–E). We estimate that ~28% of ABNs showed increased activity during the preS period (Figure 1F, top). However, event frequency returned to preC levels during the postS and test periods. Indeed, ~10% of the ABNs showed decreased activity after shock experience (i.e., preS to PostS, Figure 1F, bottom).

**Figure 2:**
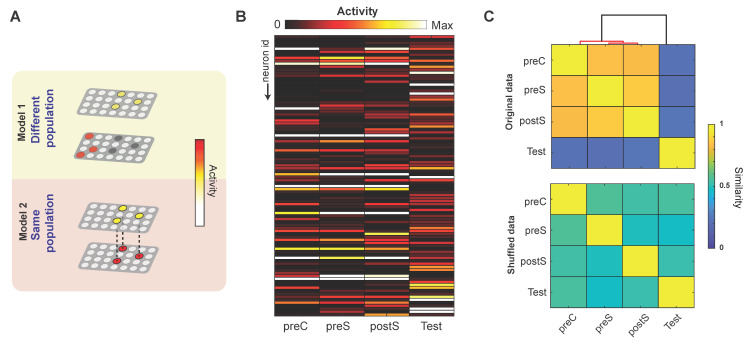
No overlap between ABN populations active during learning versus retrieval. (**A**) Two potential models by which population ABN activity could change between two different periods. (**B**) ABN activity vectors in different periods. Each row represents the mean activity of individual ABNs. Each activity vector was rescaled from 0 to 1. (**C**) Similarity matrix (i.e., cosine similarity) between pairs of activity vectors for the original data (top) and after random shuffling (bottom). The overlaying dendrogram represents the results of hierarchical clustering, with significant clusters (*p* < 0.05) shown in red. *n* = 94 neurons from 4 mice.

These observations of individual ABN activity, however, do not clarify whether a similar or different population of ABNs is recruited in different periods. For instance, a difference in activity could reflect the recruitment of a new population that is active in a specific period (Figure 2A, model 1) or a change in the activity of the same population (Figure 2A, model 2). To address this issue, we arranged the mean activities of individual ABNs into column vectors across different periods (Figure 2B, hereafter referred to as activity vectors). If a similar population of ABNs is recruited in two different periods, its activity will be correlated. We analyzed this possibility by creating a similarity matrix (i.e., cosine similarity) considering all possible comparisons between activity vectors (Figure 2C). Subsequent hierarchical clustering of this matrix revealed that a similar population of ABNs is active across the preC, preS, and postS periods, suggesting that neither novel context exposure nor shock experience recruits a different ABN population. Surprisingly, a different ABN population was predominantly active during the test period, suggesting that active ABN populations do not overlap between learning and retrieval.

**Figure 3:**
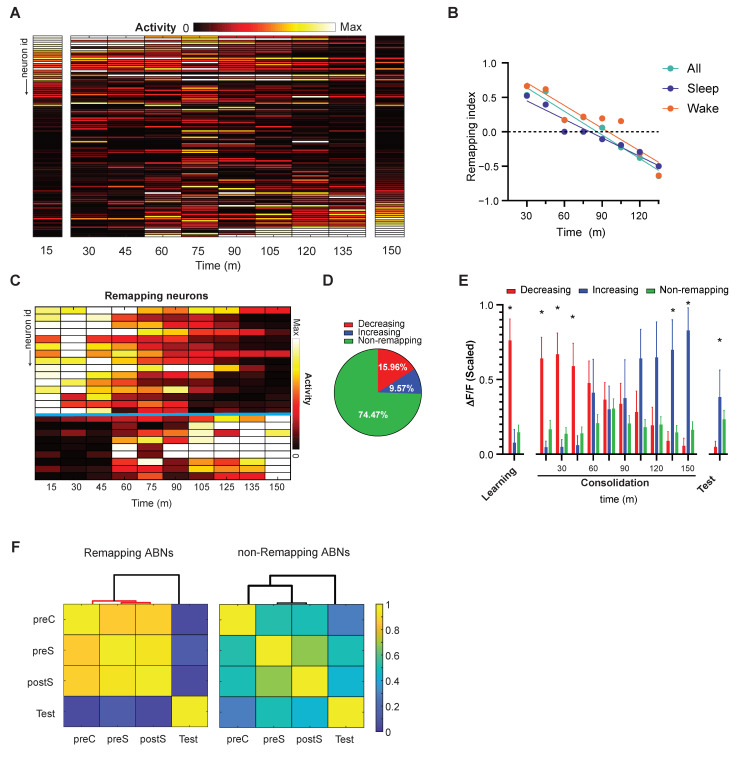
ABN activity remapping during fear memory consolidation. (**A**) Activity vectors during memory consolidation (15 min bins). To represent data in the same range, the maximum activity of each neuron was scaled to 1. (**B**) Remapping index relative to the first and last activity vectors shown in (**A**). All, r = −0.97 *p* < 0.0001 slope = −0.0114; sleep, r = −0.98 *p* < 0.0001 slope = −0.0090; wake, r = −0.93 *p* = 0.008 slope = −0.0109. (**C**) Changes in ABN activity were correlated with time (Pearson’s correlation, *p* < 0.05; adjusted by false discovery rate, *q* < 0.05). (**D**) Pie chart of ABNs showing decreasing, increasing, or no change (i.e., non-remapping) in activity over time. (**E**) Mean activity of remapping and non-remapping ABNs during learning (preS and postS, averaged), consolidation, and test periods. Means were calculated after scaling activity vectors. * *p* < 0.05 between red and blue bars (bootstrap). (**F**) Similarity matrix between remapping and non-remapping ABNs. Significant clusters (red lines; hierarchical clustering, *p* < 0.05) were found only among remapping ABNs. ABNs decreasing their activities: *n* = 15; ABNs increasing their activities *n* = 9. Non-remapping ABNs *n* = 70. Total *n* = 94 neurons from 4 mice.

This segregation of ABN population activity may take place during memory consolidation. To address this possibility, we divided the consolidation period into 15 min bins and calculated an activity vector for each bin (Figure 3A). Next, we calculated a remapping index that indicates whether a given activity vector is closer to that at the beginning or end of the consolidation period. This remapping index ranges from −1 to 1; a value of 1 indicates a perfect match to the first bin of the consolidation period, a value of −1 indicates a perfect match to the last bin, and a value of 0 indicates equidistance from both. Remapping indices were strongly correlated with time, indicating that ABN activity gradually remapped during the consolidation period (Figure 3B). This remapping was detected, regardless of whether mice were awake or asleep (Figure 3B). It was not possible to calculate remapping indices during REM sleep given the limited amount of REM sleep during the consolidation period (<10% of total time) and the sparse activity of ABNs during this sleep stage (<6% of total ABN activity occurs in REM sleep) [16].

Activity remapping may occur in all or a specific population of ABNs. To discern between these two possibilities, we traced ABNs with activities significantly correlated with time during the memory consolidation period. This revealed two subgroups of neurons, one gradually decreasing their activities, and another one increasing their activities (Figure 3C,D). The ABNs decreasing their activities were predominately active during the preS/postS periods; in contrast, the ABNs increasing their activities were predominantly active during the test period (Figure 3E). Accordingly, significant clusters in the similarity matrix were only detected for the ABNs that showed significant changes in activity during consolidation and not among those that showed no correlation (Figure 3F). Previously, we reported that the ABNs that are active during the postS period are more likely to be reactivated during REM sleep, and the ABN activity in REM sleep is necessary for memory consolidation [16]. This population of ABNs that is reactivated during REM sleep overlaps with the population of ABNs decreasing their activities during the consolidation period (Figure S1C). This suggests that reactivation during REM and activity remapping may be linked phenomena, although causality is yet to be demonstrated.

Collectively, these results suggest that the populations of ABNs that are active during fear learning and retrieval are segregated over time by an activity remapping process (graphical abstract).

## 3. Discussion

Here, we show evidence that ABN activity remaps over time during fear memory consolidation. ABN activity during learning did not reflect the recruitment of a new ABN population. In sharp contrast, the ABN population active during the test did not overlap with that active during learning. This suggests that ABNs do not hold fear memory traces in a conventional manner [19].

We also found evidence suggesting that the segregation of ABN population activity is caused by a time-dependent process of activity remapping. Neurons exhibiting decreased activity during the consolidation period are those that were predominantly active during learning. Previous models predict that ABNs segregate memories when different learning experiences are separated by time scales of days or weeks [11,12]. Thus, the remapping of ABN activity within hours suggests that memory segregation may occur faster than previously postulated.

One open question is whether the vigilance state of the animal influences activity remapping. A possible approach to address this would be to correlate ABN activities during individual sleep or wakefulness episodes with activity remapping [22]. However, this approach is unfeasible given the sparse nature of ABN firing [16]. Other important questions include whether ABN activity remapping is learning dependent and specific to immature ABNs.

In summary, our results indicate that ABN activity gradually remaps during fear memory consolidation, which leads to the emergence of different ABN populations that are active during learning versus retrieval. This activity remapping may potentially allow ABNs to segregate memories encoded a few hours apart. These results advance our understanding of the role of adult neurogenesis in the mammalian memory system.

## 4. Materials and Methods

### 4.1. Experimental Model

The data in this manuscript were obtained by reanalyzing the Ca^2+^ imaging data related to Figure 1I,J in [16]. The methods used to obtain these data are described in detail in [23].

All animal experiments were approved by the University of Tsukuba Institutional Animal Care and Use Committee. Mice were maintained in home cages in an insulated chamber with an ambient temperature of 23.5 ± 2.0 °C under a 12 h light/dark cycle with ad libitum access to food and water. Mice (Jackson Laboratory, Sacramento, CA, USA) harboring pNestin-CreER^T2^ (nestin mice, stock #016261) and Rosa26-pCAG-loxP-stop-loxP(LSL)-GCaMP3 (GC mice, Ai38, stock #014538) were backcrossed in a C57BL6/J background more than 10 times. Nestin^+/WT^ mice were bred with GC^+/+^ mice, resulting in F1 GCnestin and GCWT offspring at a nearly 1:1 ratio. Only male F1 mice were used. Mice were habituated to experimenter handling by two or three 2 min handling sessions/day for a total of 11 sessions before behavioral experiments.

To induce GC expression in ABNs, all F1 mice were treated with tamoxifen at 7 weeks of age. Tamoxifen (120 mg/kg, Toronto Research Chemicals, Toronto, Canada) was injected into the peritoneal cavity five times at 1- or 2-day intervals, with completion of the injection period within 10 days.

### 4.2. Implantation of Lens and EEG/EMG Electrodes

Surgery was performed at 9 weeks of age. The microendoscope lens (1 mm diameter, 4 mm length, Inscopix, Palo Alto, CA, USA) was placed at anterior–posterior (AP) −2.0 mm, medial–lateral (ML) +1.2 mm, and dorsal–ventral (DV) −1.95 mm. EEG electrodes were placed at AP +1.5 mm and −3 mm and ML −1.7 mm. EMG electrodes were bilaterally placed into the trapezius muscles. One week after electrode placement, the baseplate for a miniaturized microendoscope camera (nVista, Inscopix, Palo Alto, CA, USA) was attached above the implanted microendoscope lens. Mice were habituated to the attached microendoscope camera for 7–8 days before recording. Lens location was histologically verified after experiments.

### 4.3. Ca^2+^ Imaging and Analysis

Ca^2+^ imaging was performed at 11 weeks of age. We performed 6 days of habituation to microendoscope attachment before contextual fear conditioning. On the conditioning day, we attached the microendoscope to mice at ZT = ~0 and performed Ca^2+^ recording for 10 min in the home cage and an additional 10 min in context A before the foot shock during conditioning. We then detached the microendoscope (<1 min) to avoid a change in the field of view due to the mouse hitting the microendoscope against the wall during shock, performed the delayed shock protocol as previously described [16,23] in context A, reattached the microendoscope (<1 min), and performed 5 min of recording after the shock in context A. Subsequently, we performed 2.5 h of recording toward the latter part of the 5.5 h consolidation period in the home cage. Finally, we recorded for 10 min during the memory retrieval test in context A.

To extract significant Ca^2+^ transients, recording sessions were concatenated and subsequently motion-corrected in mosaic v1.2 (Inscopix, Palo Alto, CA, USA). Fluorescence traces from single neurons were extracted in MATLAB using constrained non-negative matrix factorization for microendoscopic data (CNMF-E) [24]. Specific details of the extraction of Ca^2+^ transients were previously described [23]. Ca^2+^ traces were deconvolved using CNMF-E (AR2, thresholded). In this study, only mice with at least 10 ABNs in the field of view were considered (total of 94 neurons from 4 mice).

### 4.4. Mathematical Analyses

Data were analyzed with MATLAB (Mathworks, Natick, MA, USA) using the image processing and machine learning toolboxes.

#### 4.4.1. Bootstrap Analysis

Statistical comparison of the means in Figure 1C and Figure 3E was performed by bootstrap analysis. Points were randomly sampled from the original sample to produce a surrogate sample, which was then used to obtain a bootstrap estimate of mean differences between groups. This was repeated 10,000 times to obtain a distribution of different bootstrap estimates. By defining a 1 − α confidence interval within this distribution, we tested whether differences between sample means were statistically different from 0. An α value of 0.05 was divided by the number of multiple comparisons in each experiment (i.e., Bonferroni-corrected).

#### 4.4.2. Identification of ABNs Responding to Novel Context Exposure

The mean activity of each neuron in the home cage was compared with its mean activities in preS and postS periods (Figure 1D). The statistical significance of these differences was estimated by moving block bootstrap [25]. This method differs from classic bootstrap in that blocks of data, rather than individual points, are sampled. This was performed to preserve temporal correlations in activity traces (Figure 1B). The length of the block (10 s) was estimated from the circular autocorrelation function shown in Figure 1B, which reflects the time during which ABN activities were aggregated.

#### 4.4.3. Similarity between Activity Vectors

The activity vectors in Figure 2B and Figure 3A are the mean activity of individual ABNs arranged in column vectors. To reduce the effect of possible outliers, extreme values falling outside the 95th percentile of the distribution of mean activities were truncated to the 95th percentile for each mouse. Data from each mouse were concatenated into a single activity vector. Activity vectors are shown scaled from 0 to 1. The similarity between pairs of activity vectors was estimated by cosine similarity (i.e., normalized dot product), defined as:

(1) $$\cos\left(A,B\right)=\frac{A\cdot B}{||A||||B||}$$

where *A* and *B* are two different activity vectors.

#### 4.4.4. Hierarchical Clustering

To identify clusters of activity vectors, we used agglomerative hierarchical clustering. Clusters were combined using angular distance (i.e., cosine distance) and Wards linkage. Statistically significant cut-off points in the dendrogram were estimated on the basis of a reference distribution of linkages obtained by random resampling of activity vectors (10,000 replicates) [26].

#### 4.4.5. Remapping Index

The remapping index (RI) is defined as:

(2) $$\mathrm{RI}=\frac{AD\left(A,B^{t}\right)-AD\left(C,B^{t}\right)}{AD\left(A,B^{t}\right)+AD\left(C,B^{t}\right)}\;$$

where *AD* is the angular distance, which is equal to 1 − cosine similarity value; *A* and *C* are the activity vectors from the first and last 15 min of the consolidation period, respectively; and $B^{t}$ is the activity vector at time *t*.

#### 4.4.6. Classification of Active and Inactive ABNs

ABNs were classified as active or inactive, as shown in Figure S1. This was performed by randomly resampling the activity traces of each neuron to create 10,000 replicates. A neuron was classified as active if it showed ≥1 Ca^2+^ transients in 95% of replicates.

## Data Availability

The data (raw calcium transients and activity traces) and the code supporting the finding of this study (MATLAB) are available at https://github.com/vergaloy/Remapping_ABNs (accessed on 1 February 2021).

## Supplementary Materials

The following are available online at https://www.mdpi.com/1422-0067/22/6/2874/s1, Figure S1: ABNs active during the postS period are reactivated during REM sleep and show decreased activity during consolidation.
