# Supplementary material for: Remapping of Adult-Born Neuron Activity during Fear Memory Consolidation in Mice
Source: Int J Mol Sci. 2021 Mar 12;22(6):2874. doi: 10.3390/ijms22062874 (PMC7999719; doi:10.3390/ijms22062874)
Supplement: Supplementary file 1 [file ijms-22-02874-s001.pdf]

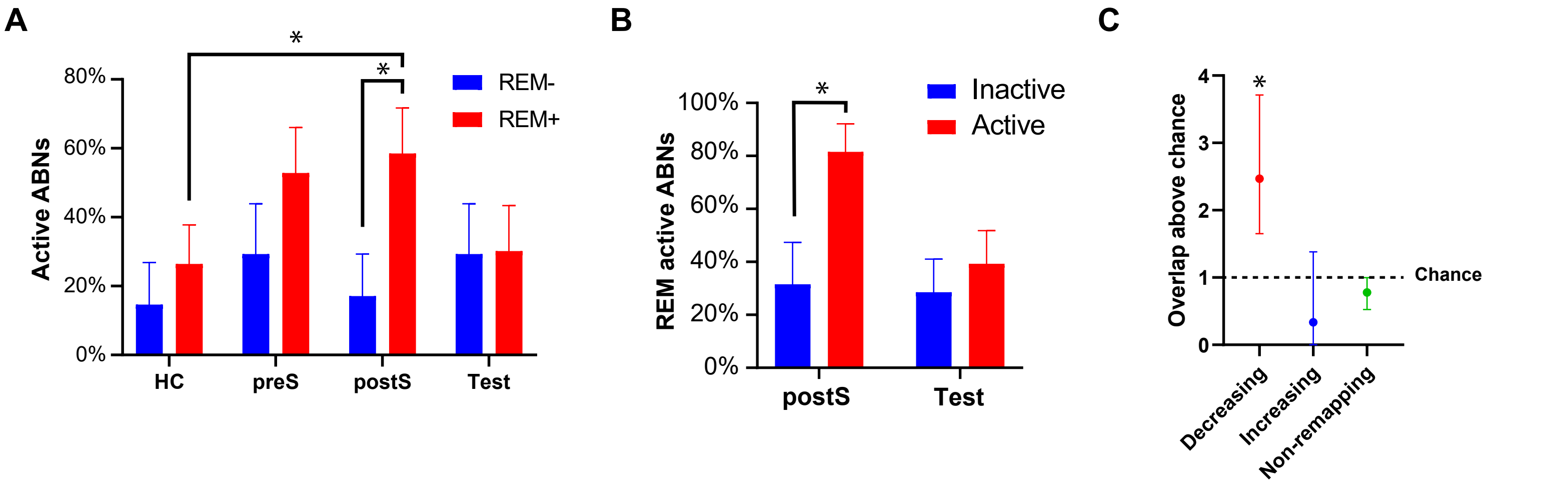

Figure S1. ABNs active during the postS period are reactivated during REM sleep and show decreased activity during consolidation. (A) ABNs were classified into REM active (REM+) or inactive (REM-) groups based on the presence of activity during REM sleep. For each group, the bar graph shows the percentage of active ABNs in each period (*$p < 0.05$, 95% confidence interval; n = 53 (REM-) and 40 (REM+) neurons). ABNs active during REM sleep were more likely to be active during the postS period as previously reported [16]. (B) ABNs were classified based on their activity during postA and test periods. For each group, the bar graph shows the percentage of ABNs active during REM sleep (*$p < 0.05$, bootstrap; error bars, 95% confidence interval; n = 56, 38, 66, and 28 neurons in postS-, postS+, test-, and test+ groups, respectively). ABNs active in the postS period were more likely to be active during subsequent REM sleep. Panels A and B correspond to Figures 1J -1K from Kumar et al. (2020). (C) ABNs decreasing their activity during the consolidation period significantly overlapped with those that were reactivated during REM sleep (i.e., ABNs that were active during postS and REM sleep).
